# Defective expression of ATG4D abrogates autophagy and promotes growth in human uterine fibroids

**DOI:** 10.1038/cddiscovery.2017.41

**Published:** 2017-08-14

**Authors:** Abdeljabar El Andaloussi, Samar Habib, Gizem Soylemes, Archana Laknaur, Heba Elhusseini, Ayman Al-Hendy, Nahed Ismail

**Affiliations:** 1 Department of Obstetrics and Gynecology, Medical College of Georgia, Augusta University, Augusta, GA, USA; 2 Clinical Microbiology Division, Department of Pathology, University of Pittsburgh, Pittsburgh, PA, USA

## Abstract

Uterine fibroids (UF) are the most common pelvic tumors in women of reproductive-age and they usually cause heavy menstrual bleeding, pain and infertility. Autophagy is a collection of processes that enables the cells to digest and recycle their cytoplasmic contents, such as toxic protein aggregates, defunct or disused organelles and invading microorganisms. Dysregulation in autophagy process were described in neoplasms; however, the contribution of autophagy to the pathogenesis of UF remains unknown. In this study, we demonstrate that autophagy is deregulated in human UF as evidenced by significant accumulation of autophagosome in human UF cells compared to normal myometrium cells. Analysis of the autophagy markers revealed an enhanced initiation of the autophagy in UF tissues compared to their adjacent myometrial tissues (MyoF). However, autophagosome maturation and flux was blocked in UF tissues, as marked by accumulation of LC3-B and P62 protein. This block was associated with defective expression of autophagy-related protein 4 (ATG4) in the UF tissues compared to MyoF in ~90% of patient samples. Silencing of ATG4D in normal human myometrial cells resulted in defective autophagy flux, enhanced cell proliferation and increased extracellular matrix production, which phenocopy UF cell line. This study indicates that impairment of autophagy flux secondary to defective expression of ATG4D expression is a new mechanistic aberration that contributes to UF pathogenesis. Targeting autophagy pathway could provide novel medical therapeutic approach for non-surgical treatment of UF.

## Introduction

Uterine fibroids (UF) are common benign monoclonal tumors, which arise from the uterine smooth muscle cells.^1^ UF causes heavy menstrual bleeding, pain, infertility, and pregnancy complications.^2^ Many studies have confirmed the role of estrogen, progesterone and other growth factors including cytokines, chemokine, and miRNA in the etiology of this disease as key regulators of their proliferative growth.^3^ The most established risk factors of UF are age, early menarche, low parity and African ancestry.^4^ However, obesity and the consistent exposure to estrogen are believed to be etiological factor that increase the incidence of UF.^1–5^ In United States, the annual health care and management cost related to UF are calculated to be up to $34 billion.^6^

Autophagy is a collection of processes that enables the cells to remove and recycle their cytoplasmic contents as toxic protein aggregates, damaged organelles, and invading microorganisms.^7^ Although the process of autophagy is regulated by more than 30 proteins, most of them are involved in the autophagosome biogenesis.^8^ These proteins are known as autophagy-related proteins (ATGs). Studies have shown that ATG4 endopeptidase activity is important for late stages of autophagosome maturation in erythroid cells and allows the fusion of autophagosomes with lysosomes.^9^ There are four orthologues of the ATG4 mammalian family, also known as autophagin. These are; autophagin-1/ATG4A, autophagin-2/ATG4B, autophagin-3/ATG4C, and autophagin-4/ATG4D. These orthologues contain all the residues required for the catalytic activity of cysteine proteases, including the conserved cysteine residue within the catalytic site.^10^ The activities of different ATG4 orthologues can be regulated by microRNAs after transcription.^11,12^ The *in vivo* model of ATG4 loss of function was done only for ATG4B and ATG4C.^13^ Animals deficient in Atg4C show an increased susceptibility to tumorigenesis.^10^ Atg4C is not essential for autophagy development under normal conditions but is required for a proper autophagic response under stressful conditions such as prolonged starvation.^14^ However, Atg4B knockout mice showed normal survival, with minimal lesions in central nervous system.^15^ The connection between human UF and autophagy has not yet been investigated. In this work we demonstrate that autophagy is deregulated in human fibroid due to lack of ATG4D induction. Autophagy induction could be a promising therapeutic strategy to treat UF and remodel abnormal fibroid tissues into normal myometrium.

## Results

### Autophagy flux is altered in human uterine fibroid

**Figure 1:**
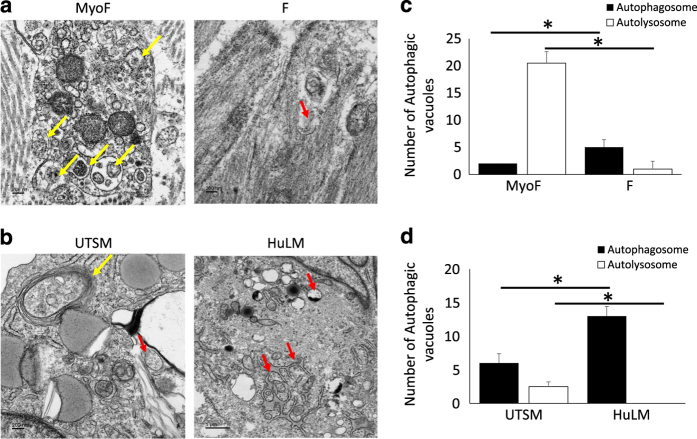
Autophagy blockade at the autophagosome stage in Human UF. Autophasome formation was analyzed* in vivo* in Fibroid tissues (F) and adjacent normal myometrium (MyoF) collected from different patients as well as in fibroid cell line (HuLM) and normal myometrial cells (UTSM) using transmission electron microscopy (TEM) as described in the Materials and Methods. Representative TEM sections from (**a**) patient biopsy and (**b**) UTSM and HuLM cell lines. (**c** and **d**) semi-quantitative measurement of the number of autophagosomes (consistent with autophagy block) and autolysosomes (consistent with autophagy flux) in patients (*n*=5) with fibroid (**c**) and in cell lines (**d**). Yellow arrows: Autolysosome (single-layer vacuole with content), Red arrows: autophagosome (double-layer vacuole). Representative images are shown (*n*=5). *P<0.05.

Autophagy is a process that leads to degradation of subcellular constituents via formation of autophagsosmes that fuse with lysosome and generate autolysosomes.^16^ We hypothesized that uncontrolled proliferation of myometrial cells and the development of fibroid could be linked to defect in the autophagy pathway. To test this hypothesis, we first evaluated different steps of the autophagy process (i.e., formation of autophagsosome and autolysosome) in human UF tissues versus adjacent matched MyoF from the same patient, using transmission electron microscopy (TEM). As shown in Figure 1, human UF tissues exhibited significantly less autolysosomes compared to adjacent myometrium. Furthermore, there was significantly more (20.5±2.12) autophagolysosome in MyoF controls compared to UF tissues (1±0.04, *P*=0.008; Figures 1a and c). This observation was further confirmed in human fibroid cell lines (HuLM) when compared to human normal myometrial cells line (UTSM) (Figure 1b). Consistent with *in vivo* data, the cell lines showed absence of autolysosomes versus control UTSM cell line (2.5±0.7) and accumulation of autophagosomes in HuLM human fibroid cell line (13±1.41) compared to UTSM (6±1.41, *P*=0.038) (Figures 1b and d). These results suggest that human fibroid phenotype is associated with normal autophagy initiation flux, but a block in autophagy flux, most likely due to a defect in the fusion of autophagosome with lysosome.

**Figure 2:**
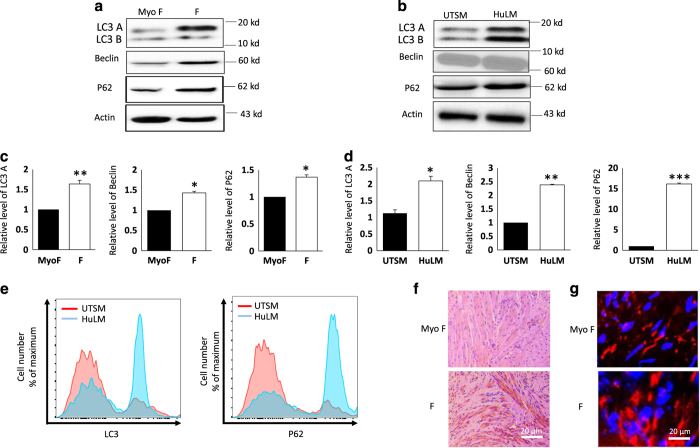
Initiation of autophagy is enhanced in Human UFs. Fibroid tissues (F) and adjacent normal myometrium (MyoF) collected from different patients, as well as in fibroid cell line (HuLM) and normal myometrial cells (UTSM) were analyzed for the expression of markers of autophagy flux by western blot. (**a**) Human UFs biopsy (MyoF, adjacent normal myometrium and F, UF). (**b**) Cell lines UTSM, HuLM. Levels of lysate proteins loaded in the gels were monitored by immunoblotting with an anti-Actin antibody. (**c** and **d**) the relative expression of LC3I/II, P62, ATG4D and Beclin1 was calculated based on the grayscale value of *β*-actin from patient biopsies and UF human cell lines respectively. (**e**) Quantification of endogenous of LC3 and P62 by flow cytometry. Histograms show a higher expression of LC3 and p62 in HuLM compared to UTSM. Data are from one experiment and representative of three independent experiments. (**f**) Immunohistochemistry staining of LC3 A/B in (MyoF) and (**f**) from UF patients (40× magnification). (**g**) Immunofluorescence staining for LC3 in human fibroid cells (magnification 63×). Representative images are shown. Data are means of three independent experiments. *P<0.05, **P<0.01, ***P<0.001.

Autophagy process is initiated in UFs. To further examine the autophagy process in human UF, we evaluated LC3 expression; a marker of autophagosome formation from the phagophore, the SQSTM1/P62 which serves as a link between LC3 and ubiquitinated substrates,^17^ and Beclin, a marker of early stage of autophagy initiation process in human UF tissues and HuLM cell lines versus MyoF tissues and UTSM cell line, respectively, by western blots. We found an increase in the autophagosome markers LC3I, LC3II, p62, and Beclin-1 in the fibroid, compared to their adjacent tissue control group from the same patient (Figures 2a and b). The observed increase in both LC3I and LC3II suggest that conversion of LC3I to LC3II by lipidation is hindered in the fibroid and points toward possible deregulation of the autophagy flux. This observation was further supported by FACS analysis of LC3 and p62 level, through intracellular staining using unconjugated antibody used for the immunoblotting (Figures 2c and d). Furthermore, this data was supported by the evaluation of LC3 and P62 by immunohistochemistry and immunofluorescence, respectively (Figures 2e and f).

### Autophagy blockade in UF is linked to defect in ATG4D

**Figure 3:**
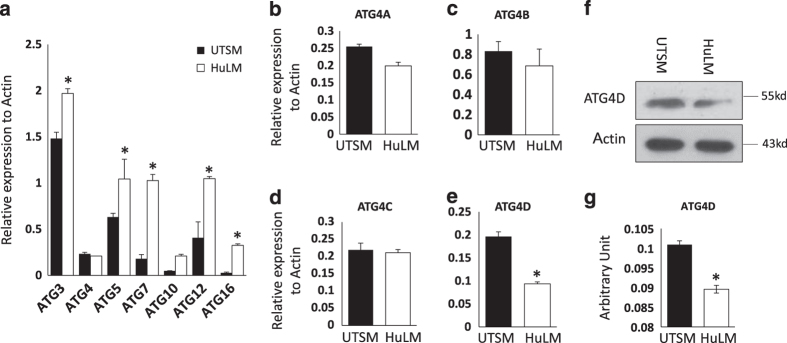
Altered expression of autophagy-related proteins (ATGs) in Human uterine fibroid. RNA was extracted from frozen myometrium tissue and reverse transcribed as described in the Materials and Methods. (**a**) Real-time PCR analysis of mRNA levels of several ATGs. Data show significantly higher expression of ATG3, AtG7, ATG12, ATG16, but not ATG4, in HuLM compared to UTSM. (**b–e**) Quantification of the ATG4D isoforms by real-time PCR showing lower expression of ATG4D isoform. (**f**,** g**) Western blot analysis of ATG4D and the expression of ATG4D normalized to Actin. Data represent mean±SEM of three independent experiments. *P<0.05.

The autophagy-related genes (ATGs) have been originally identified in the human genome and are largely associated with formation of the autophagosome membrane. We compared the expression level of several ATGs by real-time PCR between human fibroid versus myometrial cell lines. Our data demonstrated a significantly higher expression in ATG3, ATG5; ATG7, ATG12 and ATG16 in HuLM compared to UTSM cell lines (*P*<0.05; Figure 3a) which is consistent with autophagy induction in UF. Importantly, the expression of total ATG4 was not induced showing the same level in both cell lines compared to other selected and analyzed ATGs. (Figure 3a). ATG4 or autophagin is a cysteine protease that has 4 orthologues A, B, C, and D, and is pivotal for the conversion of LC3I to LC3II and formation of the autophagosome.^18,19^ Thus, we proceeded to determine their expression levels in UF versus myometrial cell lines by real-time PCR. We found a significant decrease in ATG4D mRNA level in fibroid cell line HuLM compared to the control UTSM cell line (*P*=0.005; Figure 3e). The expression of the remaining ATG4 orthologues was not significantly different between HuLM and UTSM (Figures 3b and e). To further analyze ATG4D expression at protein level, we examined the ATG4D level by western blot. Our data indicates significant decreased expression of ATG4D normalized to the level of actin in human fibroid versus myometrial cell lines (Figures 3f and g). The correlation between decreased expression of ATG4 and the conversion of LC3I to LC3II in HuLM suggest that ATG4D defect may contribute to defective fusion between autophagosome and lysosome in human fibroid lesions.

### Defective ATG4D expression in human UFs

**Figure 4:**
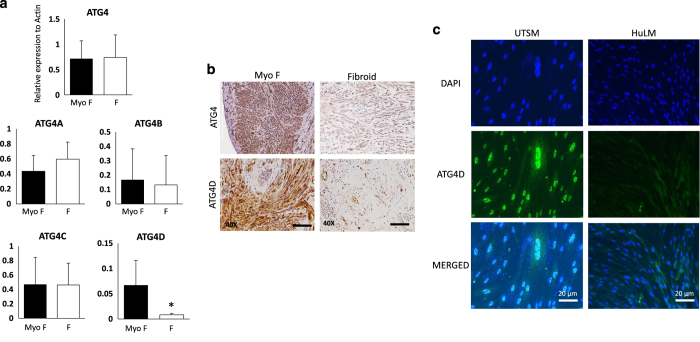
ATG4s are not induced in Human UFs patient biopsies and fibroid cell line. (**a**) Real-time PCR quantification of total ATG4 mRNA expression and ATG4 isoforms. (**b**) Immunohistochemistry staining with human anti-ATG4 and anti-ATG4D. (**c**) Immunofluorescence staining for ATG4D in UTSM and HuLM cell lines, Green ATG4D and Blue is DAPI. (*n*=15, *P< 0.05).

The autophagy process is orchestrated by many ATG in order to regulate the cellular homeostasis.^20^ To corroborate the *in vitro* data, we quantified the mRNA expression levels of several ATGs in patient UF versus MyoF biopsies. We did not detect significant difference in the expression of ATG3, ATG5, ATG7, ATG10, ATG12, and ATG16 in UF compared to MyoF tissues (data not shown). The ATG4 expression defect was noted in 90% of patients and their average show absence of expression induction in UF (Figure 4a). Our finding was supported by immunohistochemistry (Figure 4b) and immunofluorescence staining of UTSM and HuLM UF cell lines with anti-ATG4D data (Figure 4c). Together, these data suggest that defective ATG4 induction in fibroid tissues and corresponding cell line is more likely due to decreased expression of ATG4D.

### Lack of ATG4s induction and diminishment of lysosome associated membrane LAMP in UFs

**Figure 5:**
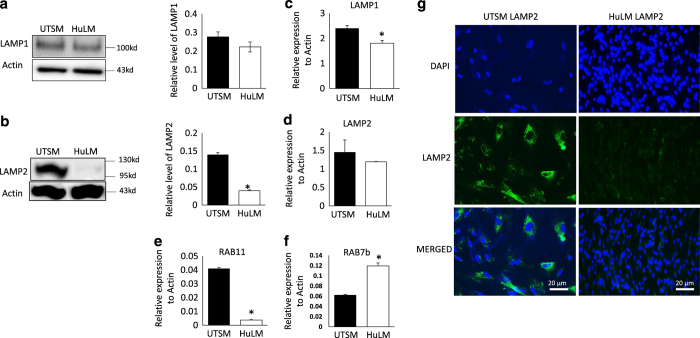
Defective expression of lysosomal markers in UFs. (**a**, **b**) Western blot analysis and semi-quantitative ratio to actin of LAMP1 and LAMP2. (**c**-**f**) Real-time PCR of LAMP1, LAMP2 and Rab11respectively. (**g**) Immunofluorescence staining for LAMP2 expression with fluorescein (green) and DAPI (blue) used to counterstain nucleus shown in single stain and corresponding merged form. (*P<0.05).

LAMP-1 and LAMP-2 deficiency in various metabolic conditions, neurodegenerative diseases and infectious diseases is linked with the accumulation of autophagosomes.^21–23^ We hypothesized that defective fusion of the lysosome and autophagosome could impair the clearance of damaged organelles and aggregated proteins in fibroid cells likely leading to increased cell proliferation. To test this hypothesis, we first analyzed the markers involved in lysosome fusion process, namely LAMP1 and LAMP2, in HuLM and UTSM cell lines by immunoblotting (Figures 5a and b) and their quantification by real-time PCR (Figures 5c–e). We found no significant change in the expression of LAMP1, however LAMP2 protein level was significantly decreased in (*P*<0.05) and clear expression induction of Rab7b (Figure 5f) but not in Rab11 significantly lower in HuLM compared to UTSM (*P*=0.00018). Furthermore, using immunofluorescence technique, there was significantly less LAMP2 expression in HULM versus UTSM cells (Figure 5g). Our data shows a second defect related to lysosome that may be involved in the observed autophagy blockade in UF.

### ATG4D silencing in normal myometrial cells generates a fibroid-like cell profile

**Figure 6:**
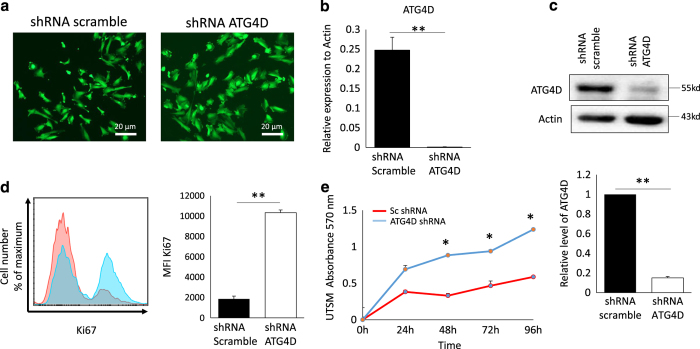
Knock-down of ATG4D by shRNA in UTSM fibroid cell line increase proliferation. The expression of ATG4D in UTSMs was knocked-down by ATG4D-specific shRNA using lentivirus particles. Control cells were infected with lentivirus containing non-specific shRNA vector. (**a**) Silencing the expression of ATG4D in UTSM by shRNA and scramble control both tagged with GFP construct. (**b**) Real-time PCR of ATG4D mRNA expression. (**c**) Western blot of ATG4D protein. (**d**) Proliferation assay analysis by FACS intracellular staining for Ki67. (**e**) The cells (3000/well) from either ATG4D knockdown or scrambled control were seeded onto 96-well tissue culture plates. The MTT assay was performed at different time points 24, 48, 72, and 96 h. The Averaged cell numbers from triplicate wells were used in preparing the showed data graph. Each data point is the mean (±S.D.) from an individual experiment performed in triplicate (*n*=3). *P<0.05, **P<0.01.

To directly investigate the contribution of ATG4D in the control of cell proliferation, and extracellular matrix production, the two hallmarks of uterine fibroids, we knockdown the expression of ATG4D in normal UTSM myometrial cell line. The ATG4D was silenced in UTSM cell line by ATG4D shRNA using scramble shRNA as a negative control. The transfected cells were selected by puromycin and the positive transfected cells were positive for GFP, a cis-cistronic marker, as well (Figure 6a). The ATG4D silencing expression was confirmed by real-time PCR and western blot as compared to scramble control (Figures 6b and c). Remarkably, loss of ATG4D expression in UTSM cells resulted in significant increase in cell proliferation compared to the scramble control cell as evidenced by significant increase in the mean fluorescence intensity (MFI) of Ki67, proliferative marker (10346 ±258.8 *versus* 1860±281.42, *P*=0.001; Figure 6d). In addition, MTT assay was performed to investigate proliferation and viability of UTSM ATG4D shRNA cells versus scramble control at different time point 24, 48, 72, and 96 h. Our data revealed a significant increase in the proliferation and viability/survival of UTSM with loss of function in ATG4D (*P*<0.05; Figure 6e). This data suggests that expression of ATG4D in myometrial cells is essential for myometrial cell proliferation and viability, and that disruption of ATG4D expression and function generate a fibroid-like cell phenotype likely via abrogation of the autophagy process.

### Loss of function of ATG4D by shRNA in normal myometrium cell line abrogates autophagy and enhances inflammation

**Figure 7:**
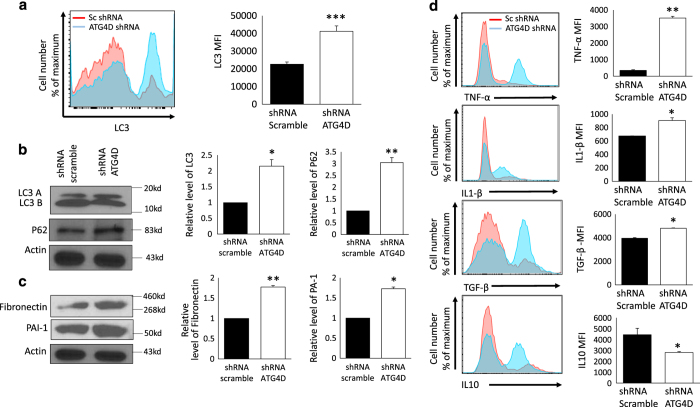
Knock-down of ATG4D by shRNA in UTSM cell line mimic UF phenotype. (**a**) Autophagy markers analysis by FACS with intracellular staining and mean fluorescence intensity MFI for LC3, and (**b**) by western blot for LC3 and P62. (**c**) Extracellular matrix markers analysis by western blot. (**d**) Endogenous quantification of pro-inflammatory cytokines expression was done by intracellular staining for TNF-*α*, IL-1*β*, TGF-*β* and IL-10 using flow cytometry analysis. The expression level was represented as mean fluorescence intensity (MFI). Data from the histogram are shown as mean±S.D. and are representative of three independent experiments. *P<0.05, **P<0.01, ***P<0.001.

ATG4D is a pivotal protein for dilapidation of LC3B from LC3A that leads to fusion of autophagosome with lysosome. We hypothesized that silencing ATG4D will block autophagy in human UTSM normal myometrial cells. To test this hypothesis, we examined accumulation of LC3A and P62 proteins by immunoblotting and flow cytometry in ATG4D-KD UTSM versus scramble control. Our data show a significant increase in the MFI of LC3I in ATG4D knockdown UTSM cells versus scramble control (*P*<0.005; Figures 7a and b). Furthermore, western blot analysis also revealed significant increase in P62 expression in ATG4D-KD UTSM versus scramble control (Figure 7b). Thus, loss of function of ATG4D in normal myometrium cell line resulted in alteration of autophagy process that phenocopied UF tissues and HuLM cell lines.

Previous studies indicated that UF pathogenesis is marked by an increase of extracellular matrix (ECM) due to excessive secretion of interstitial collagen, inflammatory immune cells infiltration and fibrosis. To examine the impact of ATG4D knockdown on ECM, we have used two markers: Fibronectin as glycoprotein of ECM and plasminogen activator inhibitor-1 (PAI-1). The analysis by western blot of extracted protein from UTSM stable cell lines treated with shRNA ATG4D showed significant increase in Fibronectin and PAI-1, compared to shRNA scramble controls (Figure 7c). This suggested that defective expression of ATG4D enhances extracellular matrix and fibrosis development. Another emerging characteristic of UF is the promotion of inflammation as reported by our group and others.^24,25^ Furthermore, recent reports connected aberration of autophagy with altered inflammatory state.^26^ In order to explore the impact of ATG4D silencing on the inflammatory response of normal human UTSM myometrial cells, we examined the expression level of proinflammatory cytokines such us TNF-a, IL-1b and TGF-b in ATG4D-KD-UTSM versus scramble control cells. As shown in Figure 7d, silencing of ATG4D expression by shRNA was able to activate proinflammatory cytokine release and enhanced inflammatory status. The intracellular staining for TGF-*β*, TNF-*α* and IL-1*β* was significantly increased in ATG4D-KD-UTSM cells compared to scramble control infected cells (*P*<0.05; Figure 7d).

## Discussion

The autolysosome pathway plays an important role in degrading and recycling different cellular components.^27,28^ Abrogated autophagy has been reported in several different diseases but not in uterine fibroid. We show here, for the first time, that autophagy is initiated in UF but blocked at the autophagosome stage. TEM analysis of UF tissues collected at different stage of menstrual cycle from patients of different ethnicity exhibited accumulation of autophagosome bilayer vacuoles, which failed to fuse with lysosomes. In contrast, autophagy flux was complete in normal myometrium tissue as evidenced by the presence of the different characteristic structures corresponding to the different steps of autophagy process beginning with the formation of autophagosome and ending with fusion of autophagsome with the lysosome to form autolysosome. The analysis of classical markers of autophagy by immunoblotting showed an accumulation of LC3I and LC3II, Beclin and P62 in patient samples as well as in cell lines; *in vitro* model of UF, supporting the TEM results. As the molecular regulation of autophagy is orchestred by ATG proteins, we evaluated the autophagic process by examining expression of ATGs. The molecular screening of several ATGs mRNA expression in UF human biopsies show high variability in their expression. However, the ATG4 expression was not induced in 90% of our patient samples. The ATG4 exist in four orthologues: ATG4A, ATG4B, ATG4C and ATG4D. Intriguingly, our data reveal that ATG4D expression was significantly decreased and almost absente at the protein level as revealed by immunohistochemistry and immunofluorescence from both patient samples as well us cell lines USTM and HuLM. ATG proteins encoded by AuTophaGy-related (ATG) genes, which have been extensively investigated in yeast, are important for autophagy process.^29^ Notably, ATG4D appears to be the most frequent ATG4 protease involved in other gynecologic cancers such as breast cancer, followed by 4B and 4C. Deregulation of ATG4 is frequent in tumors of the female reproductive system such as ovarian, uterine and breast cancers reflecting its importance in stem cells homeostasis.^14,15^ Thus, it is possible that ATG4D is the master regulator of autophagy fux in several gynecologic benign and malignant tumors. How ATG4 is regulated in these tumors is not clearly understood. However, studies have shown that ATG4 can be regulated by miR-34a that specifically targets ATG4B,^11^ miR-376b that target intracellular levels of ATG4C,^12^ and the tumor suppressor miR-101 that inhibits autophagy by targeting ATG4D.^13^ At the functional level, the biological role of ATG4D in gynecologic tumor biogenesis and progression is not well understood. However, studies using zebrafish indicated that ATG4D is critical for autophagy-mediated neuronal homeostasis in the central nervous system, where knockdown of ATG4D in neuronal cells resulted in abnormal autolysome formation and degradation and cytoplasmic vacuolization. Further studies suggested that ATG4 is responsible of LC3 delipidation at the autophagosome stage. Atg4s is expressed at low level in a wide variety of human tissues at low levels. The cleavage of Atg4D is related to its unique roles in both autophagy and apoptosis.^23^ The majority of ATG4s alterations have been detected in female reproductive tissue tumors, including ovarian and uterine cancers.^30^

UF is proliferative disease that is characterized by uncontrolled cellular proliferation, inflammatory tumor microenvironment and immature extracellular matrix. To understand and directly evaluate the role of ATG4D in the pathogenesis of UF, we knocked-down ATG4D by shRNA in normal myometrium cell line UTSM. We found that ATG4D loss of function in UTSM promotes proliferation of myometrial cells as evidenced by increased intracellular staining for Ki67 (Figure 6d) and supported by the MTT proliferation assay at different points in culture (24, 48, 72, 96 h; Figure 6e). Notably, unlike the expected decrease in cell viability upon genetic manipulation by shRNA, our data indicate that deficiency of ATG4D enhanced the survival of shRNA-treated UTSM compared to the controls (Figure 6e). These data are consistent with other studies showing that Atg4D silencing in HeLa cells sensitize them to starvation-induced cell death, suggesting that Atg4D mediated autophagy contributes to the survival response in starved cells.^31^ Consistent with the *in vivo* and *in vitro* phenotypes of UF tissues and HuLM cells, respectively, knockdown of ATG4D in UTSM also resulted in increased autophagy initiation, but block of autophagy flux as marked by increased expression of LC3 and p62 in UTSM treated with shRNA ATG4D. Further analysis of the fibronectin and PAI-1; markers of extracellular matrix, by western blot demonstrated a high expression of these ECM in UTSM in the absence of ATG4D. The induction of ECM markers correlate with the release of inflammatory cytokines such us TGF-*β*, that lead to fibrosis through structural remodeling of the myometrium. Furthermore, the mRNA expression of multiple ECM genes in UF is decreased when the TGF-*β* pathway is downregulated.^32^ Thus, based on these observations regarding autophagy in UF, our data unravel a novel mechanistic pathway in UF pathogenesis where ATG4D loss of function in normal myometriumal cells was able to induce UF phenotype. Further studies using mutant mice lacking ATG4S could be useful model to further analyze the role of this molecule in autophagy *in vivo*;^33,34^ however these mice are not currently available.

Our data indicated that the expression of LAMP2, but not LAMP1; markers of lysosomal acidification, was attenuated in both the cell lines and patient samples. It is not yet completely understood why LAMP1 was not regulated in a manner similar to LAMP2 in fibroid; however, it is possible that downregulation of LAMP2 is more critical in lysosomal acidification, fusion of autophagsome and lysosome, and thus formation of autolysosome. This possibility is supported by recent study demonstrating that deficiency of LAMP2 in mice resulted in a defect of autophagosome–lysosomal fusion, subsequent accumulation of autophagosomes in the muscles, and cardiomyopathy.^35^ In addition, the impairment of autophagosome and lysosome fusion contributes to the accumulation of autophagic vacuoles containing substrates such us toxic aggregate.^36^ These data further support our conclusion of autophagy blockade at autophagsome-lysosomal fusion stage. Further studies will examine the intriguing link between defective Atg4D expression and autophagy blockade in fibroid.

In conclusion, the present study suggests that the impairment of autophagy is a causative factor of UF formation primarily due to lack of ATG4D expression induction. Our results provide a novel mechanistic aberration that are significantly involved in UF pathogenesis and propose ATG4/ATG4D (lack of induction) as new biomarker associated with UF growth. Further investigations are needed to evaluate pro-autophagy compounds for possible novel therapeutic against UF able to induce autophagy and clear abnormal proliferative cells by apoptosis.

## Materials and methods

### Patients’ characteristics

All UF samples were collected from intramural fibroids 3–8 cm in size and adjacent myometrium (at least 2 cm from fibroid capsule). To minimize patient-to-patient variability, all our tissue samples were collected in proliferative (*n*=10), secretory (*n*=2) and inactive phase (*n*=3) phase of menstrual cycle (based on dates and confirmed by histological examination) and from African American women. From each patient the UF and normal myometrium control were collected. The resected tissues were collected under pathologist supervision where samples were provided to the research team promptly removed from the subject. The tissues were rinsed in cold phosphate-buffered saline (PBS) three times and were either flash frozen and stored at −80°C, for nucleic acid preparation, or were immediately digested for primary cell isolation. Details of patient characteristics are summarized in Table 1. 

### Cell lines and cell culture

Uterine smooth muscle cell line (UTMS) and human fibroid (HuLM) are immortalized human cell lines generously provided to us by Dr Darlene Dixon (National Institute of Environmental Health Sciences, Research Triangle Park, NC, USA). The cells were cultured in DMEM medium supplemented with 10% FBS at 37°C in 5% CO_2_ atmosphere. Adherent cells were trypsinized using 0.25% trypsin-EDTA and re-plated into flasks every 2–3 days. Cells in the logarithmic phase of growth were used in subsequent analyses.

### Protein extraction and western blot

The cells were lysed in 100 *μ*l of lysis buffer (RIPA/PMSF). The samples were incubated on ice for 30 min, the cell lysate was centrifuged at 18,000×g for 8 min at 4 °C, and the supernatant was collected. The protein concentration in the supernatant was quantified using the BCA kit (23225) purchased from Thermofisher Scientific (West Columbia, SC, USA). The proteins were then separated by SDS-PAGE and transferred onto a nitrocellulose membrane. The blocking treatment of the membrane was done by bovine serum albumin and the membrane was incubated at 21 °C overnight with human anti-actin (dilution, 1 : 250), human anti-LC3A/B (dilution, 1 : 250), human anti-P62 (dilution, 1:250), human anti-ATG4D (dilution, 1 : 250), human anti-Beclin1 (dilution, 1 : 250), human anti-fibronectin (dilution, 1:500) and human anti-PAI (dilution, 1 : 500) monoclonal primary antibodies. Subsequent to washing, the mouse anti-rabbit and rabbit anti-human secondary antibodies were added at dilutions of 1 : 10 000 and 1 : 5000, respectively, and the membrane was incubated at 21 °C for 4 h. Finally, the membrane was developed by using an enhanced chemiluminescence reagent (34076) purchased from ThermoScientific (Waltham, MA, USA). The developed film was scanned using the Alpha Imager 2200 gel imaging system. The relative expression of anti-LC3B (2775) and anti-Beclin1 (D40C5) from Cell signaling. Anti-P62 (ab56416), anti-LAMP1 (ab24170), anti-LAMP2 (ab25631), anti-ATG4D (ab137621), anti-Fibronectin (ab2413), and anti-LAM2 from Abcam (Cambridge, MA, USA). Anti-PAI was purchased from Peprotech ((Rocky Hill, NJ, USA) 500-P260) was calculated based on the grayscale value of *β*-actin (dilution, 1:1000) and purchased from Sigma (St Louis, MO, USA, 55414).

### Measurement of cell proliferation

MTT assay is a colorimetric assay using a dimethylthiazoldiphenyltetra- zoliumbromide (M 5655) purchased from Sigma, for the nonradioactive quantification of cellular proliferation and viability. The cells (3000/well) from either ATG4D knockdown or scrambled control were seeded onto 96-well tissue culture plates purchased from Becton Dickinson. The MTT assay was performed at different time points 24, 48, 72 and 96 h. The Averaged cell numbers from triplicate wells were used in preparing the data graph. Each data point is the mean (±S.D.) from an individual experiment performed in triplicate (*n*=3). The plate reading was done using the Biotek Gen5 software (Winooski, VT, USA).

### RNA isolation and real-time PCR

RNA was extracted from frozen myometrium tissue by using Invitrogen TRIzol reagent (15596026) according to the manufacturer’s instructions. A total of 1–5 *μ*g of total RNA was reverse transcribed using Superscript III reverse transcriptase from Invitrogen to prepare cDNA. Real-time PCR was performed by using iTaq Universal SYBR Green Supermix from Bio-Rad (Raleigh, NC, USA). The primers sequence of the following genes are described in Supplementary Table S1. The following cycles were performed: an initial denaturation cycle of 94 °C for 5 min, followed by 40 amplification cycles of 94 °C for 15 s and 60 °C for 1 min on Bio-Rad qPCR machine. The results are represented as expression relative to actin.

### Silencing ATG4D expression

Lentiviral shRNA (Origen Technologies Inc., Rockville, MD, USA) targeted to autophagy-related protein-4D (ATG4D) RNA was used to knockdown ATG4D protein expression. For infection, lentivirus particles were added to each well of a six-well plate containing 1×10^5^ cells. Control cells were infected with lentivirus containing non-specific shRNA vector. Cells were incubated with lentivirus for 12 h and next transferred to a 75 mm flask. Infected cells were selected following treatment with puromycin (1 *μ*g/ml) and based on positivity for GFP expression. The ATG4D knockdown cell lines showed reduced expression level compared to scramble control. Cells treated with scramble shRNA vector are hereafter referred to as autophagy-competent. ATG4D protein knockdown cells are hereafter referred to as autophagy blocked.

### Histology and immunohistochemistry

Patient MyoF and UF tissues were collected, fixed in 4% paraformaldehyde, and embedded in paraffin. Tissue sections were stained with haematoxylin-eosin (H&E) staining as well as with human anti-ATG4 (abx111110) from Abbexa Ltd (Cambridge, UK), anti-ATG4D (ab137621), anti-LC3 antibodies (D3U4C) from Cell Signaling as mentioned above, followed by detection with a biotin-labeled rabbit anti-rat antibody and staining with the ABC kit purchased from Vector Laboratories. The samples processing and staining was done using standard techniques.^13^ The slides analyses were performed using Olympus Las 4.1 software (Center Valley, PA, USA).

### Immunofluorescence

Patient samples were embedded in paraffin and processed in the histology core facility of Augusta University using standard techniques.^13^ The biopsy samples were fixed with 4% paraformaldehyde. After permeablization by 0.3% Triton X-100 and incubated with goat serum, cells were stained with appropriate antibody for overnight at 4 °C. Then, cells were incubated with a secondary antibody at 37 °C for 1 h and DAPI (0100-20) purchased from Southern Biotech (Birmingham, AL, USA) for 10 min. Finally, samples were examined with a Nikon confocal microscope (Nikon C1-Si, Tokyo, Japan).

### Transmission electron microscopy analysis

The cell samples was fixed and prepared as described previously.^14^ Serial ultrathin sections were cut on an LKB-III ultratome from LEICA, and were stained with uranyl acetate and lead citrate both of them purchased from TED PELLA. The sample sections were examined using a Hitachi H7600 electron microscope at an accelerating voltage of 100 kV, at the histology core facilities of Augusta University using standard techniques.^13^ The counting methods of autophagosome and autolysosome vacuoles was done based on TEM sections as described previously.^37^

### Flow cytometry and cytokines staining

The intracellular staining for autophagy and inflammation markers was done after fixation and permeabilisation of cells (554714) using BD Cytofix/Cytoperm purchased from BD Bioscience according to the manufacturer’s protocol. The anti-human marker for autophagy LC3 (D3U4C) that was conjugated with anti-rabbit IgG Fab2 (4414S), purchased from Cell signaling and P62 (ab56416) that was conjugated with Goat DyeLight 488 IgG (ab96879) purchased from abcam. Proliferation marker Ki67 (350514) and anti-human proinflammation cytokine markers such us TNF-*α* (502943), TGF-*β* (349607) and IL-10 (506804) were purchased from Biolegend except IL-1*β* (IC8406A) that was purchased from R&D. The intracellular staining for cytokines was done in the presence of GolgiSTOP (554715) purchased from BD Biosciences. 1×10^5^ cell line cells were incubated with the Abs for 30 min at room temperature. The cells were washed twice with PBS/2% FBS (v/v) and resuspended. Samples were acquired and analyzed using a BD Bioscience FACSCanto as well as FlowJo software for data analysis.

### Statistical analysis

Statistical comparison of differences between the two test groups was evaluated by one-way ANOVA test or Student’s *t*-test analysis as appropriate. A *P*-value of less than 0.05 was considered statistically significant. All values are expressed as mean±S.E. and analyzed using Prism Graph-Pad Software Inc (La Jolla, CA, USA).

## Publisher’s note

Springer Nature remains neutral with regard to jurisdictional claims in published maps and institutional affiliations.

## Figures and Tables

**Table 1:** Patients’ data

*Patient #*	*Age*	*BMI*	*Race*	*Endometrium*
P1	41	29.7	AA	Proliferative
P2	46	30.3	AA	Proliferative
P3	45	37.6	AA	Proliferative
P4	43	32.9	AA	Proliferative
P5	34	29.4	White	Proliferative
P6	44	41.8	AA	Proliferative
P7	44	31.4	AA	Proliferative
P8	45	40	AA	Proliferative
P9	54	28	AA	Proliferative
P10	43	37.6	White	Proliferative
P11	42	38	White	Inactive
P12	43	35	White	Inactive
P13	54	38.5	AA	Inactive
P14	37	55.2	AA	Secretory
P15	45	31.1	AA	Secretory

Abbreviations: AA, African-American; BMI, body mass index; P, patient.
