# Supplementary material for: Defective expression of ATG4D abrogates autophagy and promotes growth in human uterine fibroids
Source: Cell Death Discov. 2017 Aug 14;3:17041–. doi: 10.1038/cddiscovery.2017.41 (PMC5554887; doi:10.1038/cddiscovery.2017.41)
Supplement: Supplementary Information [file cddiscovery201741-s1.pdf]

Suplementary table 1: Primers sequence

Primer Name	Sens 5' ----- 3'	Anti-sens 3'----- 5'
Actin	CGTCATTGCACGAAGACACAA	CCTGGTCCACCATTTTAAGGC
ATG3	ACATGGCAATGGGCTACAGG	CTGTTTGCACCGCTTATAGCA
ATG4	CCAGCCCACTGTGGATGTC	AAGCCCACGGTACAGCTTG
ATG4A	TTCTTGGACCCTCATACAACCC	TTAGGATGTTCATTCGCTGTGG
ATG4B	GGTGTGGACAGATGATCTTTGC	CCAACTCCCATTTGCGCTATC
ATG4C	TAGAGGATCACGTAATTGCAGGA	GTTGTCAAAGCTGAGCCTTCTAT
ATG4D	TATGGGCCATCGCTAGTGG	CATACACGGGGTTGAGAGTCT
ATG5	AAAGATGTGCTTCGAGATGTGT	CACTTTGTCAGTTACCAACGTCA
ATG7	ATGATCCCTGTAACTTAGCCCA	CACGGAAGCAAACAACTTCAAC
ATG10	AGACCATCAAAGGACTGTTCTGA	GGGTAGATGCTCCTAGATGTGAC
ATG12	CTGCTGGCGACACCAAGAAA	CGTGTTCGCTCTACTGCCC
ATG16	TCTGGGACATTCGATCAGAGAG	CCTTTCTGGGTTTAAGTCCAGG
LAMP1	TCTCAGTGAACTACGACACCA	AGTGTATGTCCTCTTCCAAAAGC
LAMP2	TGGCAATGATACTTGTCTGCTG	ACGGAGCCATTAACCAAATACAT
